# Novel Material Optimization Strategies for Developing Upgraded Abdominal Meshes

**DOI:** 10.3390/ijms241814298

**Published:** 2023-09-19

**Authors:** Alfred Najm, Adelina-Gabriela Niculescu, Marius Rădulescu, Bogdan Severus Gaspar, Alexandru Mihai Grumezescu, Mircea Beuran

**Affiliations:** 1 Department of Surgery, Carol Davila University of Medicine and Pharmacy, 050474 Bucharest, Romania; alfred.najm@yahoo.ro (A.N.); bogdangaspar2005@yahoo.com (B.S.G.); drmirceabeuran@yahoo.com (M.B.); 2 Emergency Hospital Floreasca Bucharest, 014461 Bucharest, Romania; 3 Research Institute of the University of Bucharest—ICUB, University of Bucharest, 050657 Bucharest, Romania; adelina.niculescu@upb.ro; 4 Department of Science and Engineering of Oxide Materials and Nanomaterials, Politehnica University of Bucharest, 011061 Bucharest, Romania; 5 Department of Inorganic Chemistry, Physical Chemistry and Electrochemistry, Politehnica University of Bucharest, 011061 Bucharest, Romania; radulescu_marius@yahoo.com; 6 Academy of Romanian Scientists, Ilfov No. 3, 050044 Bucharest, Romania

**Keywords:** abdominal meshes, mesh materials, polymer materials, composite materials, optimization strategies, improved biomedical devices

## Abstract

Over 20 million hernias are operated on globally per year, with most interventions requiring mesh reinforcement. A wide range of such medical devices are currently available on the market, most fabricated from synthetic polymers. Yet, searching for an ideal mesh is an ongoing process, with continuous efforts directed toward developing upgraded implants by modifying existing products or creating innovative systems from scratch. In this regard, this review presents the most frequently employed polymers for mesh fabrication, outlining the market available products and their relevant characteristics, further focusing on the state-of-the-art mesh approaches. Specifically, we mainly discuss recent studies concerning coating application, nanomaterials addition, stem cell seeding, and 3D printing of custom mesh designs.

## 1. Introduction

Abdominal wall hernias are common defects caused by the weakening and loss of continuity of the fasciae and/or muscles with the protrusion of visceral organs, representing an important source of disability and morbidity in patients [1,2,3,4]. More than 20 million hernias are operated annually around the globe, with most surgical procedures imposing the need for mesh implantation [5,6,7].

**Figure 1:**
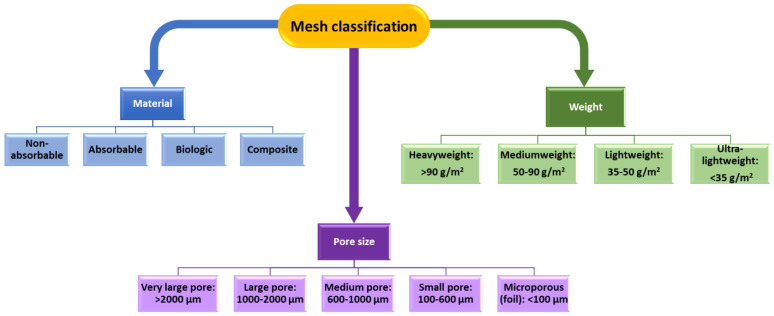
Mesh classification according to material, pore size, and weight. Created based on information from [11].

Abdominal meshes were introduced to improve traditional suturing, which tends to bring excessive tension in the affected area, high recurrence rates, and increased pain [1,3]. The advances in the interconnected fields of health services, medical technology, and material sciences have led to the fabrication of numerous hernia repair meshes. Products made of different polymers with varied physicochemical properties (Figure 1) are currently available on the market for surgeons to choose from when bridging defects or reinforcing the abdominal wall [1,8,9,10].

However, despite the many available options, certain challenges remain to be solved for developing improved reinforcement devices. Some of the remaining issues include repair failure, dysfunctional healing, and mesh-related complications [1,12]. Thus, mesh development represents an ongoing process [8], with new formulations being under investigation by scientists worldwide.

In this context, this paper aims to review the most common materials used for manufacturing abdominal meshes, mentioning the commercially available devices for each polymer. Even though mesh products have been previously described in other works [1,11,13,14,15,16], this review provides an updated framework on the topic, emphasizing the recent developments. Moreover, an extensive discussion is offered on the newest material optimization strategies that reportedly led to superior mesh characteristics. In more detail, we mainly analyzed studies describing coating application, nanomaterials addition, stem cell seeding, and 3D printing of custom mesh designs. For this section, we have reviewed English language publications from the last 5 years retrieved from Science Direct and Google Scholar databases to offer a cutting-edge perspective on mesh development to serve as an inception point for further research in the field.

## 2. Materials Commonly Employed in Mesh Fabrication

Since the introduction of meshes in hernia treatment, various biomaterials have been considered for fabricating these medical devices to ensure proper reinforcement of the weakened area [17,18]. The material choice is crucial, being linked not only to the mechanical properties of the implant but also to the functional and histological outcomes [19]. Factors that must be considered when deciding upon the mesh material include its biocompatibility, bioreactivity, physicochemical properties, porosity, degradation rates, ease of fabrication, and costs [18,20,21]. With many commercial products already on the market, deciding upon the most suitable mesh depends on each patient’s needs, surgeon preference, and hospital stock [22].

Over the years, several absorbable and biological materials have been developed and utilized for soft tissue reinforcement, yet non-absorbable polymers are still the base materials for most meshes used in hernia treatment [7,17,23,24]. In this respect, the most employed synthetic polymers for mesh fabrication are described in the following sections.

### 2.1. Polypropylene

Polypropylene (PP) started being used as a prosthetic material in 1958. Since then, it has become a prevalent polymer for meshes, revolutionizing surgery for the repair of abdominal wall hernias and being broadly recognized as superior to primary suture repair [18,25,26]. PP meshes have, for a long time, been successfully utilized in clinical practice due to a series of advantageous properties [27]. This material exhibits relative biological inertness, high tensile strength, and good in situ longevity [28]. High-density PP was also reported as less susceptible to infection than other materials [26], being a more favorable option to nylon or polyethylene terephthalate for repairing abdominal wall defects after infected wound dehiscence [28]. Moreover, PP meshes are pliable, allow connective tissue infiltration, and ensure robust mechanical support [2,26]. PP mesh flexibility can be altered by either using knitted or woven materials, whereas filament structure was noted to influence pain levels. Specifically, patients treated with multifilament meshes exhibited lower pain scores and faster return to work than patients treated with monofilament meshes [26,29].

Despite their appealing features, PP meshes have also been correlated with several undesired consequences. It is known that the organism responds to mesh implantation through an inflammatory reaction, further inducing scar plate formation and increased abdominal wall stiffness. Other unwanted effects assume adhesion to visceral organs, mesh incorporation or encapsulation, biomaterial shrinkage, and decreased mechanical properties [2,24,25]. Moreover, a new study by Wang et al. [30] revealed that, despite being used as a non-absorbable mesh material, PP was found to degrade in vivo, contradicting its supposed biostability characteristics. Nevertheless, the correlation between PP degradation and postoperative complications has not yet been completely elucidated.

To counteract these side effects, a wide range of PP-mesh coating methods have been proposed and developed [24]. Yet, PP fiber coating is not always the best solution. A recent study by Ayuso et al. [31] reported higher rates of postoperative wound and mesh infection following open preperitoneal ventral hernia repair when using coated PP meshes (i.e., Ventralight and Proceed) than with uncoated prosthetics, recommending the utilization of an uncoated mesh for extraperitoneal placement. 

An alternative strategy for reducing foreign body response gained more ground in the clinical setting. Namely, reducing PP density and producing lightweight meshes was highly employed for improving abdominal wall compliance, diminishing mesh contraction or shrinkage, enhancing tissue incorporation, and reducing both postoperative and chronic pain [2,25,26,32]. 

A series of PP-based meshes are currently used in practice, either as the sole component or in combination with other materials, including, but not limited to, collagen [33], sodium hydrogel [34], oxidized regenerated cellulose and polydioxanone [35], polyglactin 910 [14,36], and polyvinylidene fluoride [37]. For an at-glance perspective, Table 1 briefly summarizes the market available meshes containing PP.

### 2.2. Polyethylene Terephthalate Polyester

Polyethylene terephthalate (PET) is a commonly used synthetic biomaterial for tension-free abdominal wall hernia repair, yet it is less frequently employed than PP [26,40]. As a polyester, PET is non-toxic, biocompatible, stable in vivo, and nondegradable, possessing required mechanical properties while displaying only a minimal inflammatory reaction by the organism [20,41,42,43,44]. Its high malleability, good mechanical strength, non-absorbability, and durability in physiological conditions render PET one of the best candidates for biomedical devices that must be left in the body for extended periods, promoting increased adaptation and patient comfort [41,42,43,45,46,47].

Polyester meshes present minimal stiffness, minimal shrinkage, minimal adhesion formation, and excellent tissue ingrowth and integration profiles [23]. Abdominal wall augmentation can be ensured through either warp-knitted or nonwoven PET fiber textile structures [45,48].

Nonetheless, despite its appealing bulk properties, PET electrospun nanofibers were noted to generate a foreign body reaction, which is also associated with considerable inflammation with tissue ingrowth into the interstices of the mesh. Moreover, PET meshes’ microporous, braided-fiber architecture raises concerns about infections, fistulas, and bowel obstructions [23]. Given that the chemical base is the same, the main causes behind the inflammatory reaction were concluded to be the reduced diameter of the fibers and the pore size of the implanted meshes [26,43,45]. To minimize these unwanted effects, manufacturers started applying various compounds in the form of coatings onto PET meshes [26].

Table 2 briefly synthesizes relevant aspects of PET-based market available meshes.

### 2.3. Polytetrafluoroethylene

Polytetrafluoroethylene (PTFE) is another frequent material choice for fabricating medical textiles. This synthetic fluoropolymer of tetrafluoroethylene was discovered by DuPont in 1938, but only started being used for hernia repair in the 1950s. However, PTFE exhibits small pores that hinder molecular permeation, provoking poor clearance of fibrinous and proteinaceous materials and eventually leading to complications, such as postoperative seroma [26].

In the 1960s, modifications were made to expand PTFE to create a uniform structure with improved mechanical properties, known as e-PTFE [26]. e-PTFE soon became a better and more popular alternative to the original DuPont material. Being flexible and smooth, e-PTFE provides suitable features for mesh fabrication. Moreover, it is easy to handle during elective surgery, enabling a safe and feasible option to repair and fix large diaphragmatic hernias [50]. According to the study of Ünek et al. [22], e-PTFE synthetic meshes offer durable long-term results in repairing incisional hernia, ventral hernia, and abdominal wall defects of various etiopathologies.

The commercially available PTFE and e-PTFE-based meshes have been summarized in Table 3.

Another derivative material of PTFE is its condensed form (c-PTFE), with two meshes being fabricated with this polymer: MotifMESH (Proxy Biomedical, Galway, Ireland) and Omyra^®^ mesh (Aesculap AG, Tuttlingen, Germany) [26]. A small prospective series of c-PTFE ventral hernia repairs concluded that these meshes are a valuable tool for treating patients with clean-contaminated and contaminated ventral hernias [53].

### 2.4. Poly (Lactic Acid)

Poly (lactic acid) (PLA) is another versatile polymer used in biomedical engineering. It is a biodegradable material that can be easily synthesized from renewable sources. Its appealing features include good processability, biocompatibility, slow degradation rate, and excellent mechanical properties [54,55,56,57,58]. Given its similar biomechanical characteristics with healthy intracavitary tissue and its ability to promote tissue integration, PLA has been rendered a good candidate for mesh production [56].

Yet, several drawbacks have also been reported, including low cell adhesion, poor thermal stability, and acid degradation by-products [57,58]. To overcome these limitations, PLA can be blended with other polymers to obtain new composite materials with enhanced properties, creating biomedical fixation devices for function restoration of impaired tissues [57,59] (Table 4).

### 2.5. Poly (Glycolic Acid)

Poly (glycolic acid) (PGA) possesses a similar chemical structure to PLA, yet its characteristics are very different. For instance, PGA exhibits a higher distortion temperature and higher gas barrier properties [54,58]. PGA’s thermal and mechanical properties make it an important biopolymer for medical applications, with the high molecular weight polymer version being the proper PGA form for providing adequate mechanical stability [62]. Moreover, its biomedical suitability is also supported by the ease of PGA degradation by hydrolysis followed by bulk erosion and compatibility to through metabolic pathways [58]. 

Thus, PGA is a remarkable biocompatible material with tremendous importance in clinical applications, yet, compared with PLA, it is more expensive [62]. In this context, several PGA-based meshes have been made available on the market, as described in Table 5.

### 2.6. Poly-4-Hydroxybutyrate

Another synthetic polymer relevant for mesh production is poly-4-hydroxybutyrate (P4HB). This material is fully resorbable, degrading into native collagen in 12–18 months while possessing similar strength and flexibility to permanent synthetic polymers [64,65]. These favorable properties allow increased repair strength during tissue remodeling [66]. Moreover, this polymer preserves its efficiency for soft tissue reinforcement even in infected wounds [67]. This aspect may be attributed to the unique antimicrobial and anti-inflammatory properties of P4HB [66].

Concerning hernia repair, two mesh configurations have been produced by Bard Davol (Warwick, RI, USA) from P4HB: Phasix^TM^ (bare, macroporous textile) and Phasix™ ST Mesh (combined with a resorbable hydrogel layer) [64]. As a woven monofilament mesh, P4HB maintains its tensile strength for at least 6 months, which is a long enough period for the wound strength to reach a maximum healing stage before the material loses its integrity [67]. The bare Phasix mesh has a knitting pattern similar to the classic PP mesh from Bard Davol and also possesses comparable mechanical properties before implantation [68]. Regarding Phasix™ ST Mesh, its additional hydrogel layer is comprised of sodium hyaluronate, carboxymethylcellulose, and polyethylene glycol, a fully resorbable formulation. This hydrogel acts as a barrier, separating the mesh structure from viscera and consequently minimizing tissue adherence of the bowels to the underlying P4HB construct, while the uncoated side of the mesh permits tissue ingrowth for repairing the abdominal wall.

### 2.7. Comparison

As various materials have been tackled over the years for mesh fabrication, surgeons now have a wide range of products to choose from when deciding on the best approach for repairing abdominal defects. Despite being all suitable for creating effective fixation textiles, the discussed synthetic polymers have very different physicochemical properties, as summarized in Table 6. These features must be considered when designing new meshes to adapt the porosity and textile configuration to each material toward optimizing the properties of the medical device to match hernia repair requirements. 

Moreover, meshes should be constructed to support adequate tissue reinforcement before they degrade, exhibiting a longer biodegradation period than the time necessary for tissue regeneration. Thus, in addition to the tabulated materials, composites based on polymer blends have emerged as promising alternatives. In particular, combining synthetic polymers with natural polymers resulted in structures with high biocompatibility and good mechanical properties. In more detail, synthetic polymers usually present strong mechanical properties, gradual or no biodegradation, and low biocompatibility, while natural polymers benefit from excellent biocompatibility and low immunogenicity but biodegrade too soon and display poor mechanical properties [56].

Collagen is one of the most common choices among potential natural polymers for mesh fabrication. Being the main component of natural ECM, collagen is endowed with inherent excellent physical and chemical properties that make it suitable for numerous wound healing and tissue engineering applications. One of the main advantages of collagen is its ability to promote cell growth, whereas an important drawback is its poor long-term durability [56,77,78,79,80]. Thus, in mesh fabrication, collagen has been used as an additional component to synthetic polymers, as is the case of Parietene DS (PP/collagen) [33] and Parietex (PET/collagen) [49] meshes from Covidien-Medtronic, which are covered on one side with a hydrophilic absorbable collagen film.

Another natural polymeric material of interest for mesh fabrication is silk fibroin (SF), a protein extracted from silkworm cocoons that has been long used as a tissue suture. It benefits from remarkable physical and chemical properties, such as biocompatibility, hydrophilicity, spinnability, and appropriate mechanical properties for supporting tissue regeneration. Particularly, it is considered suitable for hernia defect reconstruction due to its demonstrated capacity to strengthen the abdominal wall tissue by acting as connective tissue [56]. One bioengineered silk-based mesh has been fabricated by Sofregen Medical (Framingham, MA, USA) under the name SERI^®^, getting FDA clearance in 2013. However, this surgical scaffold has not been commercially available since 31 December 2021 [81]. Hence, despite its appealing properties, SF is yet to be more deeply researched for providing new constructs for hernia repair. 

## 3. Material Optimization Strategies

As each mesh material has advantages and disadvantages, ongoing research focuses on optimizing these medical devices through various strategies. Scientists worldwide have investigated methods for endowing implants with antimicrobial, anti-adhesion, and anti-inflammatory properties while maintaining the required biomechanical characteristics. Depending on their approach to optimizing meshes, the identified studies have been included in the following categories: coating, nanomaterials, stem cells, 3D-printed designs, and other strategies. 

### 3.1. Coatings

One highly investigated method for material optimization is applying various coatings on pre-existent meshes. For instance, Pérez-Köhler et al. [82] have developed an antibacterial mesh coating from a carboxymethylcellulose gel loaded with rifampicin. Comparing coated and uncoated meshes in *Staphylococcus aureus* and *S. epidermidis* in infected rabbits, the authors observed that rifampicin-carboxymethylcellulose gel-coated textiles could fully clear bacteria while ensuring optimal tissue integration, whereas uncoated implants displayed macro/microscopic signs of infection and impaired tissue integration. Given the encouraging results obtained with this antibiotic, the same research group [83] tackled the loading of rifampicin in a thermo-responsive hydrogel formulation to be applied after implantation. This new alternative coating method prevented implant infection, being a strong prophylactic tool to be considered in mesh-supported hernia surgery. Alternatively, Dydak and colleagues [84] proposed the use of a different antibiotic-based formulation. The researchers evaluated a bacterial cellulose polymer coupled with gentamicin as an absorbent layer for surgical meshes. The as-modified hernia meshes were reported effective in preventing infections through bacterial growth inhibition while providing excellent biocompatibility toward fibroblast cells.

On a different note, Guillaume et al. [85] focused on optimizing PP/titanium meshes by applying a novel coating based on stromal vascular fraction (SVF) combined with fibrin. The SVF-coated meshes were noted to influence angiogenesis levels in the early stages of tissue healing, but longer-term studies are required to check how this effect correlates with more robust mesh integration compared to non-coated implants.

Various polymeric coatings have also been proposed for PP meshes. For instance, Yang et al. [86] constructed an electrospun regenerated silk fibroin (RSF) coating and compared RSF-coated meshes, polycaprolactone (PCL)-coated meshes, and uncoated meshes. The comparison revealed better inflammatory responses and antiadhesion fractions for the coated meshes than bare PP textiles. Moreover, RSF coatings provided complete peritoneal regeneration, resulting in lower IL-6 levels and higher VEGF, IL-10, and TGF-β levels compared to the PCL-coated meshes. The obtained results recommended RSF as a promising coating for promoting the regeneration of peritoneal and abdominal wall tissues. Differently, Qiao and colleagues [6] created an innovative coating comprised of a buildup of dopamine-mediated zwitterionic poly(sulfobetaine methacrylate) (PSBMA). Two strategies were approached for PSBMA coating application, both involving polydopamine (PDA) adhesive: sequential deposition (PSBMA-PDA-PP) and co-deposition (PSBMA@PDA-PP). PSBMA addition decreased the hydrophobicity of the PP mesh surface, improved its protein resistance, and attenuated foreign body reaction while maintaining good stability and adequate mechanical properties. In particular, PSBMA-PDA-PP displayed an enhanced ability against macrophage adhesion and proliferation and exhibited considerably diminished levels of TNF-α and IL-6 as compared to PSBMA@PDA-PP, demonstrating its valuable potential for coating biomedical implants. Another polymer-based coating was developed by Sanbhal et al. [87]. The authors fabricated chitosan crosslinked and levofloxacin hydrochloride-loaded antimicrobial PP mesh devices. The as-modified meshes displayed great antimicrobial properties, with inhibition zones up to 10 mm, and were able to sustain antimicrobial effects for 6 days against *S. aureus* and *E. coli*, rendering them potential candidates for contaminated/infective surgical fields. 

Serafim et al. [88] suggested one more coating-based optimization strategy. They coated commercially available PP meshes with a thin hydrogel layer of gelatin methacryloyl (GelMA) and mucin methacryloyl (MuMA). Moreover, the meshes were pre-treated with platelet-rich plasma to additionally stimulate cell interactions through the high concentration of growth factors. The as-designed coated meshes could modulate fibroblast response on implantable textiles, with GelMA generating the best cellular response. Thus, functionalized gelatin holds promise for developing upgraded bioactive meshes for hernia repair.

### 3.2. Nanomaterials

The unique features of nanodimensional materials associated with their reduced sizes and specific morphologies have been increasingly explored for a wide range of applications in medical research [89,90,91], being also the object of study of several works on meshes.

For example, Afewerki et al. [92] designed multifunctional bactericidal nanofibers as an advanced material for hernia repair meshes. Made of polycaprolactone methacrylated fibers functionalized with GelMA, the obtained material showed bactericidal activity, low inflammatory response, good biodegradation, tunable mechanical properties, good biointegration, blood vessel formation, and tissue ingrowth, offering a promising new perspective for the healing of abdominal wall defects.

A different research group [93] used the electrospinning method to create a double-layered nanofibrous membrane combining PCL, graphene oxide, and chitosan. To expand the biological functions of the membrane, the researchers also added *N*-acetylcysteine to help in repairing full-thickness abdominal wall defects. When tested in rat models, this nanostructured patch exhibited excellent mechanical strength, exceptional biocompatibility, and good anti-hernia and anti-adhesion effects, and is considered a prospective tool for abdominal wall defect reconstruction and a promising postoperative anti-adhesion agent.

Another nanofibrous structure was proposed by Liu et al. [94], who electrospun PCL and SF, further integrating the fibers with multi-walled carbon nanotubes loaded with amoxicillin. This complex nanotechnological approach generated a functional mesh with undeformed structure, biocompatible surfaces, modified interface hydrophilicity, similar mechanical properties to the abdominal wall, and sustained antimicrobial activity against *E. coli* bacteria. Moreover, its subcutaneous implantation in a rat model revealed less mesh-induced inflammatory and foreign body responses than PCL/SF mesh, and less infiltration of granulocytes and macrophages within 14 days.

Differently, Fernández-Gutiérrez et al. [95] endowed PP meshes with antibacterial properties by coating them with a film of chitosan incorporated with randomly dispersed poly(d,l-lactide-*co*-glycolide) (PLGA) nanoparticles encapsulating chlorhexidine or rifampicin. Employing the nanocarrier systems resulted in high activity against *S. aureus* and *S. epidermidis*, with strong inhibition of bacteria growth in the surrounding environment and resistance against bacterial adhesion to the mesh surface. Therefore, the proposed material formulation could be used for the prophylactic coating of PP meshes for hernia repair.

Alternatively, Whelove et al. [40] conjugated gold nanoparticles into PET meshes as a way to improve their biocompatibility. Studies revealed that this method provides enhanced cellularity, reduced ROS, and reduced bacteria adhesion to PET fibers, demonstrating that gold nanoparticle addition offers a better biocompatibility profile when compared to pristine PET meshes.

A more recent study by Giuntoli et al. [18] focused on developing a nanostructured biomimetic implant for enhanced cell attachment and new tissue formation. In this sense, the authors fabricated a multicomponent hernia mesh device by applying a nanofibrous membrane made of a PCL-gelatin blend onto commercial PP meshes. The as-designed meshes exhibited chemical and topographical cues similar to the native ECM ones, an aspect that can be exploited for improving the biological response and mesh integration for upgrading abdominal wall hernia repair. 

### 3.3. Stem Cells

In recent years, the introduction of cellular components (stem cells in particular) on meshes has started being explored as a new alternative to enhance implant biocompatibility, improve tissue integration, and minimize adverse effects [96,97].

For instance, Dong et al. [98] fabricated a composite scaffold through the combination of PLA with a thermo-responsive hydrogel (i.e., poly (*N*-isopropylacrylamide)-block-poly (ethylene glycol)) and seeded it with rat adipose-derived stem cells (ADSCs). The 3D matrix offered a high surface area/volume ratio, exceptional biocompatibility, adequate mechanical strength, ability to stimulate native ECM, and capacity to accelerate cell adhesion and proliferation. The ADSCs’ addition enhanced defect repair and regeneration, promoting early vascularization in the electrospun scaffolds. Therefore, it can be expected that the newly developed ADSCs-seeded scaffold will be of good use in restoring abdominal wall defects in the future.

Recently, Laursen et al. [99] elaborated on PCL meshes coated with connective tissue growth factor (CTGF)/PEG-fibrinogen (PF) and rat mesenchymal stem cells. The as-designed meshes were reported to offer the required support and biocompatibility without inducing long-term mesh-related complications in an abdominal repair model. The meshes were implanted in elderly female rats (mimicking elderly, post-menopausal women), making them especially relevant for pelvic floor repair research. However, the authors concluded that long-term intervention studies in a large animal model are essential before moving to human clinical practice.

Alternatively, a study by Franklyn et al. [100] evaluated muscle-derived stem cells’ effect in regenerating the anterior abdominal wall to treat iatrogenically created ventral hernia in a rat model. The cells were seeded onto PP/PCL meshes, creating an overall construct able to repair hernias with fewer adhesions and a regenerated muscle layer. Thus, this cell-based composite is a suitable candidate for laparoscopic/complex abdominal wall repairs for abdominal wall defects.

On a different note, Hansen and colleagues [101] comparatively evaluated several types of PCL-based meshes: hollow fiber PCL loaded with basic fibroblast growth factor (bFGF), solid fiber PCL with and without bFGF, and solid fiber PCL carrying connective tissue growth factor (CTGF) and rat mesenchymal stem cells (rMSC). In this study, solid PCL-CTGF mesh delivering rMSC was the only device that did not result in multiple complications, demonstrating improved biomechanical and biochemical properties and providing proper reinforcement to the weakened abdominal wall. 

Another stem cell-based strategy was proposed by Marinaro et al. [24]. The researchers seeded human menstrual blood-derived mesenchymal stromal cells (MenSCs) on multilayered fibrin-coated PP meshes. The coating ensured MenSC viability and adhesion while avoiding any change in their stemness and inflammatory profile. The incorporated cells were able to considerably decrease CD4+ and CD8+ T-cell proliferation, correlated with a reduction in inflammatory response after mesh implantation. Yet, an additional multidisciplinary, translational perspective is needed to optimize these medical devices before they enter the clinical setting.

### 3.4. 3D-Printed Designs

In addition to the material, the fabrication technique also influences the properties of the mesh [7]. The most common architecture for these devices is the knitted structure, with the majority of meshes being manufactured through the wrap-knitting process [14,102]. Morpho-structural characteristics such as pore size, thickness, surface texture, tensile strength, and flexural rigidity highly depend on the knitting pattern (i.e., the direction of courses (rows) and wales (columns) of the fiber/yarn in relation to each other). During the knitting process, fibers are curved to create a meandering path, resulting in a more flexible and elastic structure than woven fabrics and endowing the device with the capacity to adapt to the movement of the human body [14]. However, wrap-knitted meshes display greater ultimate load values and no adaptation to the anisotropic mechanical behavior of normal human abdominal wall tissues [102]. Electrospinning is an alternative fabrication method that has gained recent popularity and is especially employed for generating nanofibers. Electrospinning represents a relatively easy laboratory polymer processing technique, providing scaffolds with a large surface area-to-volume ratio and interconnected pores. Despite its versatility, cost-efficiency [103], and effective control of fiber topography and orientation, electrospinning is limited by poor mechanical properties and ineffective pore-structure controllability [104].

In this context, additive manufacturing techniques have gathered increasing scientific interest in medical device production. Particularly, promising optimization perspectives can be envisaged by fabricating meshes using 3D printing techniques. In contrast to knitting and electrospinning processes, 3D printing offers precise control over relevant surgical mesh properties, including pore size, shape, and fiber thickness, also allowing the combination of polymeric materials with drugs [56]. Herein, several studies reported on the production of 3D-printed mesh designs with improved biomechanical performance, including adequate mechanical properties, reduced inflammation, lowered adhesion, sustained drug delivery, and personalized morphological characteristics. 

As an example, Song et al. [105] recently created a PLA mesh composited with 3D printing of acellular dermal matrix material as a novel device for repairing abdominal wall defects. This new mesh displayed a smaller adhesion score than the pristine PLA one, also providing a lower inflammatory response at the contact surface between the meshes and the abdominal organs, diminished levels of IL-6 and IL-10, and increased expressions of tissue regeneration-related factors, vascular endothelial growth factor and transforming growth factor β. Thus, it was concluded that as-designed meshes are effective tools in reducing postoperative inflammation while successfully promoting abdominal wall defects repair.

Qamar et al. [106] used 3D printing fabrication to realize personalized PP and polyvinyl alcohol (PVA) meshes loaded with ciprofloxacin hydrochloride. These drug-impregnated textiles possessed satisfactory mechanical properties, with the PVA constructs displaying a slightly faster release rate than PP meshes. The use of printed meshes produced no signs of implant rejection, with mild to moderate adhesions to the viscera. Moreover, drug loading resulted in fewer fluctuations in body temperature and faster wound healing in tested animals.

A different 3D-printed mesh option was reported by Calero Castro et al. [107]. The researchers developed drug-doped PCL meshes containing alginate and gentamicin whose effects were studied in vitro in *E. coli* cultures and in vivo in rats. The meshes were observed to possess bactericidal activity and good histopathological behavior.

Recently, Olmost-Juste et al. [108] 3D-printed tailored meshes made of alginate and waterborne polyurethane. The scientists were able to create implants with patient-specific morphological characteristics through computer-aided design mesh model adaptation. Moreover, a calcium chloride coating was applied after printing as an additional reinforcement element. The optimum amount of alginate was reported to be 6 wt%, a formulation that provides a tensile strength value of 16 N cm^−1^ and adequate elasticity to cover physiological corporal movements.

One more 3D printed mesh design was offered by Ballard and colleagues [109], who fabricated custom PCL constructs impregnated with iodinated, gadolinium, and barium contrast agents. The printed meshes displayed excellent visibility on computed tomography (CT), with the barium-embedded mesh maintaining its visibility after 7 days of incubation on agar at human body temperature. These scaffolds hold promise for application in various highly personalized and CT-visible medical devices.

### 3.5. Other Strategies

Several other strategies were found in the literature and considered relevant to the topic without fitting into any of the categories described above. Thus, in this section, we reviewed different interesting studies employing less common mesh materials, innovative polymer blends and composite structures, and new implant designs.

Saiding et al. [110] constructed a PLGA heat-shrinkable electrospun fibrous tape (HS-EFT). Through the contraction of the polymer, the relaxed fascia is pulled back at body temperature, whereas the mechanical force of retraction becomes an external signal for regulating the fibroblast behavior. HS-EFT was able to reduce wound area and ensure tissue repair in herniated rabbits, providing inspiration for the structural and functional reconstruction of relaxed soft tissue in a non-invasive manner.

Li and colleagues [111] proposed a combination of poly(l-lactide-co-caprolactone) and porcine fibrinogen (PLCL/F-Fg) and compared it with a PP mesh for the repair of a canine abdominal wall defect model. At a 4:1 PLCL:F-Fg1 blend ratio, the developed meshes exhibited optimal shrinkage rate, mechanical strength, porosity, and super-hydrophilic properties. These 3D nano-network architectures possessed favorable biodegradation and biomechanical profiles after implantation, displaying an equilibrium between material degradation and host tissue in-growth that ensured proper tissue remodeling and reconstruction. 

In a study by Liu et al. [112], a functional mesh was obtained from PCL, SF, and micronized decellularized human amniotic membrane (HAM). The PCL/SF/HAM medical devices provided a superior substrate for cell proliferation and vasculogenic network than meshes without HAM, while also being capable of inhibiting transforming growth factor β1 (TGF-β1) expression and collagen secretion under inflammatory conditions. These favorable properties were further correlated with lower adhesion, weaker inflammatory response and foreign body reaction, more pronounced neovascularization, and improved incorporation of collagen, elastin fibers, and contractile filaments. Hence, the authors concluded that their newly fabricated constructs could expand the intraperitoneal applicability of electrospun meshes for compliant remodeling in repairing abdominal wall defects. 

Differently, Mori da Cunha et al. [113] incorporated ureidopyrimidinone (UPy) moieties into an aliphatic polycarbonate (PC) backbone. This new material behaved partly better than ultralight PP meshes and offers a longer degradation period than PCL. Moreover, the UPy-PC implants were replaced by a connective tissue stiff enough to prevent abdominal wall herniation in more than 60% of the gap-bridged full-thickness abdominal wall defects. Nonetheless, the outcomes were considered suboptimal when testing in rabbit models, as more vigorous inflammatory responses were noted than for PP meshes, and signs of muscle atrophy and intramuscular fatty infiltration were observed.

Chalony and colleagues [114] focused their research on creating an adhesion-free, biocompatible, nonwoven material. In this respect, they used poly (ethyl-2) cyanoacrylate reinforced by polyurethane core to generate an electrospun composite mat. The authors reported the structure is suitable for medical devices, displaying adequate mechanical properties for intraperitoneal hernia mesh implants and the ability to attenuate biological elements for repairing the viscera layer. 

Another core-shell electrospun structure was offered by Zhou et al. [115]. The researchers developed a functional “inner–outer” medicated fibrous membrane with RGD on the surface for suppressing exogenous inflammation and puerarin in the core for accomplishing long-term endogenous inflammation inhibition. The RGD was also noticed to enhance biocompatibility, promoting cell viability, adhesion, and proliferation. The fibrous membrane was demonstrated effective in a rat abdominal wall hernia model, being able to lower both exogenous and endogenous inflammation while stimulating wound healing via collagen deposition, smooth muscle formation, and vascularization.

Minardi et al. [116] created biomimetic meshes for ventral hernia repair based on type I collagen/elastin crosslinked blend (CollE). CollE was formulated both as flat sheets and porous scaffolds, with both architectures providing immediate repair of the hernia defect, promoting tissue restoration in only 6 weeks, and enhancing neovascularization through their elastin component. However, CollE formulated into scaffolds exhibited more similar characteristics to native tissues and could induce higher gene expression of the entire marker genes tested than CollE sheets. Given their appealing bioactivity and mechanical features, the as-designed meshes represent promising candidates for ventral hernia repair.

A different solution for abdominal wall defect repair is provided by Rong et al. [117]. The authors manufactured a woven cotton fabric modified with gentamicin that could be combined with a commercially available PP mesh to generate a two-layer composite structure for abdominal wall defect repair. The antibiotic-impregnated cotton fabric exhibited favorable biocompatibility and satisfactory anti-infection properties, with a bactericidal rate of over 99.99% against *E. coli* and *S. aureus*. Thus, the two layers could work in synergy, demonstrating superior results than PP meshes in preventing infectious complications in abdominal wall defect repair.

One more bilayer system was reported by Lanzalaco et al. [2], who combined a PP mesh with a covalently bonded poly(*N*-isopropylacrylamide) (PNIPAAm) hydrogel. Cell adhesion was modulated by varying parameters such as the duration of PNIPAAm grafting time, crosslinker content, and temperature of material exposure in PBS solutions. The construct had outstanding stability in dry or wet media, demonstrating the good adhesion between the thermosensitive hydrogel and the polymer surface and holds promise for expanding abdominal hernia repair options with new anti-adherent meshes.

Hu et al. [27] also directed their research efforts toward creating an anti-adhesion gel-mesh. The scientists blended a dopamine-functionalized polysaccharide derivative (oxidized-carboxymethylcellulose-g-dopamine) with carboxymethyl chitosan to form a hydrogel in situ at the appropriate time. They applied the gel on a PP mesh during laparoscopic surgery to repair an abdominal wall defect in a piglet model, effectively preventing abdominal adhesions. In addition, combining the hydrogel with the commercial mesh alleviated the inflammatory response and collagen deposition around the mesh without affecting mesh-abdominal wall integration. 

Recently, Amato and colleagues [118] thought of a different way to improve hernia repair. Specifically, the research group created a tentacle-shaped mesh designed as a central body with integrated radiating arms. The as-described device was implanted in the preperitoneal sublay, with the “tentacles” delivered across the abdominal musculature with a needle passer. The procedure was considered fixation-free as the mesh was held in place by the friction of the straps passing through the abdominal wall. In the long-term, a very low rate of complications was observed, with no recurrence being reported as well as a great reduction in pain. Thus, the researchers concluded that their tentacle system was an easy, fast, and safe method for the fixation-free repair of Spigelian hernias.

### 3.6. Overview

Searching for an ideal mesh, researchers have managed to create numerous device alternatives to currently used products. Scientific efforts have been directed toward enhancing biocompatibility, improving mechanical properties, generating multifunctional structures, and obtaining better biological responses from the host. In this respect, several strategies have gained increasing popularity, counting coating application, fabrication of composite structures, and nanotechnology involvement among the most investigated possibilities. Other promising mesh optimization approaches include seeding meshes with stem cells, electrospinning novel polymer blends, and designing customizable implants through advanced fabrication techniques. 

Table 7 better summarizes the recent findings in the field of mesh material optimization.

## 4. Conclusions and Future Perspectives

**Figure 2:**
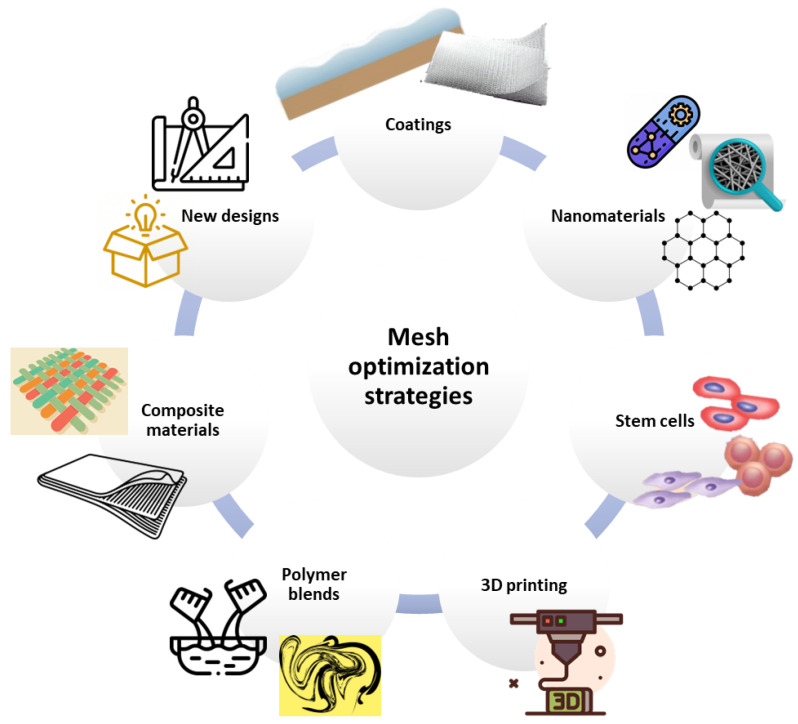
Main directions in mesh optimization strategies.

To summarize, a broad spectrum of meshes are available on the market, with the vast majority comprising of non-absorbable synthetic polymers. Nonetheless, continuous efforts are invested in developing upgraded implants by modifying existing products or fabricating completely new structures from scratch. Numerous studies have been identified in mesh material optimization, with the most common strategies for obtaining better-performing textiles outlined in Figure 2. 

Alone or used in various combinatorial approaches, the mentioned material enhancement strategies have generated promising structures for hernia repair, with optimal mechanical properties, minimal inflammatory reaction, antimicrobial activity, and low visceral adhesion. However, there is room for improvement, especially as the emerging mesh formulations have been mostly tested on small animal models. Hence, it is vital to extend the studies and explore in more depth the proposed strategies before introducing new products on the market.

In addition, multidisciplinary studies could bring unprecedented results, expanding the knowledge in the field and enriching the mesh portfolio with superior items, paving the way for personalized treatments. For instance, a promising future perspective is the integration of 3D/4D printing with artificial intelligence and machine learning for designing patient-specific implants [119]. Through ingenious material engineering, mesh structures with adjustable and gradual mechanical properties should be developed. Hence, advanced devices can be created that provide flexible and stiff regions to address the complex biomechanical behavior of the abdominal wall. 

Another interesting manufacturing perspective implies the use of embroidery technology. This method allows for creating custom-made designs in which thread direction can be arranged at almost any angle, with minor effort in pattern realization and machine adjustments compared to warp-knitting. Although not yet utilized for hernia meshes, embroidery technology has shown potential in fabricating tissue-engineered scaffolds [102,103]. Thus, it can be expected that mesh production would tackle this strategy for generating better-controlled designs. 

Moreover, future research should also be oriented to the economic aspects of the prototypes, considering the material’s availability, manufacturing costs, and financial outcomes. Specifically, a proper balance between costs and benefits must be maintained to ensure market adoption of novel meshes [12,97].

In conclusion, mesh fabrication faces promising ongoing progress, with numerous studies focusing on overcoming the challenges associated with available products. Therefore, high hopes are raised for new hernia repair solutions to be included in the surgeons’ options for treating the millions of patients in need worldwide.

## Figures and Tables

**Table 1:** Market available meshes containing PP. Created based on information from [14,15,33,34,35,36,37,38,39].

Commercial Name	Manufacturer	Technical Specifications				
		Composition	Pore Size (mm)	Weight (g/m^2^)	Filament Type	Tensile Strength (N/cm)
Prolene	Ethicon	PP	0.8	80–100	Monofilament	156.5
Marlex	Bard Davol	PP	0.8	80–100	Monofilament	58.8
3D Max	Bard Davol	PP	0.8	80–100	Monofilament	124.7
Surgipro	Autosuture/Covidien-Medtronic	PP	0.8	80–100	Multifilament	Longitudinal direction: 41.8 Transverse direction: 52.9
Prolite	Atrium/Pierson Surgical	PP	0.8	80–100	Monofilament	138
Trelex	Meadox	PP	0.8	80–100	Multifilament	n.r.
Parietene	Covidien-Medtronic	PP	0.8	80–100	Monofilament	Longitudinal direction: 38.9 ± 5.2 Transverse direction: 26.6 ± 4.2
Prolene Light	Covidien-Medtronic	PP	1.0–3.6	36–48	Monofilament	20
Optilene	B-Baun	PP	1.0–3.6	36–48	Monofilament	58
Parietene DS	Covidien-Medtronic	PP/collagen	1.5 × 1.7	73	Monofilament	42
Sepramesh	Genzyme/Bard Davol	PP/sodium hydrogel	1–2	102	Monofilament	n.r.
Ventralight ST	Bard Davol	PP/sodium hyaluronate/carboxymethylcellulose/polyethylene glycol/poly (glycolic acid)	n.r.	<40	Monofilament	n.r.
Proceed	Ethicon/Johnson & Johnson	PP/oxidized regenerated cellulose/polydioxanone	2	60	Monofilament	56.5
Vicryl	Ethicon/Johnson & Johnson	PP/polyglactin 910	0.4	56	Multifilament	Longitudinal direction: 78.2 ± 10.5 Transverse direction: 45.5 ± 13.5
Vypro	Ethicon/Johnson & Johnson	PP/polyglactin 910	>3	25	Multifilament	16
Vypro II	Ethicon/Johnson & Johnson	PP/polyglactin 910	>3	30	Multifilament	16
Ultrapro	Ethicon/Johnson & Johnson	PP/poliglecaprone 25	>3	28	Monofilament	55
DynaMesh-IPOM	DynaMesh	PP/polyvinylidene fluoride	1–2	60	Monofilament	Longitudinal direction: 11.1 ± 6.4 Transverse direction: 46.9 ± 9.7
Composix EX Dulex	Bard Davol	PP/e-PTFE	0.8	Light	Monofilament	n.r.
TiMESH extralight	pfm medical titanium gmbh	PP/titanium	>1	16	Monofilament	12
TiMESH light	pfm medical titanium gmbh	PP/titanium	>1	35	Monofilament	47

n.r.—not reported in the literature.

**Table 2:** Market available meshes containing PET. Created based on information from [11,14,15,49].

Commercial Name	Manufacturer	Technical Specifications				
		Composition	Pore Size (mm)	Weight (g/m^2^)	Filament Type	Tensile Strength (N/cm)
Mersilene	Ethicon/Johnson & Johnson	PET	1.0–2.0	33–40	Multifilament	19
Parietex	Covidien-Medtronic	PET/collagen	1.5 × 1.8	38	Multifilament	n.r.

n.r.—not reported in the literature.

**Table 3:** Market available meshes containing PTFE. Created based on information from [11,14,15,51,52].

Commercial Name	Manufacturer	Technical Specifications				
		Composition	Pore Size (mm)	Weight (g/m^2^)	Filament Type	Tensile Strength (N/cm)
Teflon	DuPont	PTFE	n.r.	n.r.	n.r.	n.r.
Dulex	Bard Davol	e-PTFE	Up to 0.5	n.r.	n.r.	n.r.
Gore-Tex	W.L. Gore and Associates	e-PTFE	0.003	n.r.	Multifilament	Minimum 16
DualMesh	W.L. Gore and Associates	e-PTFE	0.003	Heavy	Foil	Minimum 16
DualMesh Plus	W.L. Gore and Associates	e-PTFE	0.022	Heavy	Foil	157.7
Soft Tissue Patch	W.L. Gore and Associates	e-PTFE	1.3	Heavy	Nonwoven	n.r.
Composix EX Dulex	Bard Davol	PP/e-PTFE	0.8	Light	Monofilament	n.r.

n.r.—not reported in the literature.

**Table 4:** Market available meshes containing PLA. Created based on information from [60,61].

Commercial Name	Manufacturer	Technical Specifications				
		Composition	Pore Size (mm)	Weight (g/m^2^)	Filament Type	Tensile Strength (N/cm)
Tigr Matrix	Novus Scientific AB	Copolymer of glycolide, lactide, and trimethylene carbonate knitted with a copolymer of lactide and trimethylene carbonate	>1	n.r.	Multifilament	n.r.
ProGrip	Covidien-Medtronic	PET or PP mesh with PLA microgrips	1.8 × 1.8	n.r.	Monofilament	n.r.

n.r.—not reported in the literature.

**Table 5:** Market available meshes containing PGA. Created based on information from [14,15,63].

Commercial Name	Manufacturer	Technical Specifications				
		Composition	Pore Size (mm)	Weight (g/m^2^)	Filament Type	Tensile Strength (N/cm)
Dexon	Syneture/American Cyanamid	PGA	0.75	56	Multifilament	n.r.
Safil	B.Braun	PGA	0.75	56	Multifilament	n.r.
Gore-Bio-A	W.L. Gore and Associates	PGA/trimethylene carbonate	n.r.	n.r.	Membrane	n.r.

n.r.—not reported in the literature.

**Table 6:** Comparison of several physicochemical properties of discussed synthetic polymers.

Properties	Materials					
	PP	PET	PTFE	PLA	PGA	PHB
Melting temperature, °C	160–180	255	325–335	140–180	220–230	168–182
Tensile strength, MPa	21–40	47	14–31	50–70	60–99.7	40.0
Density g/cm^3^	0.900–0.915	1.39	2.16	1.2–1.4	1.50–1.71	1.18–1.26
Flexural strength, MPa	40	118	n.r.	92–100	222	n.r.
Flexural modulus, GPa	1.5	4	0.275–0.750	3.4–5	7.8	n.r.
Young’s modulus, GPa	1–1.6	3.5	1.5–1.6	2.4	7	n.r.
Elongation at break, %	80–350	2–83	70–600	5	16.4	n.r.
References	[28,54,69]	[54,70]	[71,72,73,74,75]	[54,55]	[54,62]	[55,76]

n.r.—not reported in the literature.

**Table 7:** Summative table.

Optimization Strategy	Description	Key Findings	Ref.
Coating	Meshes coated with an SVF-fibrin layer	Influenced angiogenesis in the early stages of tissue healing	[85]
Coating	Meshes coated with RSF	Improved inflammatory response and antiadhesion fractions Provided complete peritoneal regeneration	[86]
Antibacterial coating	Meshes coated with a carboxymethylcellulose gel loaded with rifampicin	Could fully clear *S. aureus* and *S. epidermidis* Allowed optimal tissue integration	[82]
Antibacterial coating	In situ application of a rifampicin-loaded thermo-responsive hydrogel	Prevented implant infection	[83]
Antibacterial coating	Meshes coated with a bacterial cellulose polymer layer loaded with gentamicin	Prevented implant infection Provided excellent biocompatibility	[84]
Antibacterial coating	Meshes coated with a levofloxacin hydrochloride-loaded chitosan layer	Displayed great antibacterial activity against *S. aureus* and *E. coli*	[87]
Coating + adhesive	Meshes coated with dopamine-mediated zwitterionic PSBMA and PDA adhesive	Improved protein resistance Attenuated foreign body reaction Exhibited enhanced ability against macrophage adhesion and proliferation	[6]
Coating + nanoparticles	Meshes coated with a film of chitosan incorporated with PLGA nanoparticles encapsulating chlorhexidine or rifampicin	High activity against *S. aureus* and *S. epidermidis* Displayed resistance against bacterial adhesion to the mesh surface	[95]
Coating + pretreatment	Meshes priorly treated with platelet-rich plasma and coated with a thin hydrogel layer of GelMA and MuMA	Could modulate fibroblast response	[88]
Coating + stem cells	Meshes coated with CTGF/PEG-fibrinogen and rat mesenchymal stem cells	Provided the required support and biocompatibility	[99]
Coating + stem cells	Meshes coated with fibrin multilayers, seeded with human MenSCs	Attenuated the inflammatory response	[24]
Bactericidal nanofibers	Polycaprolactone methacrylated fibers functionalized with GelMA	Exhibited bactericidal activity Attenuated the inflammatory response Stimulated blood vessel formation	[92]
Nanofibrous structure	Double-layered nanofibrous membrane comprising PCL, graphene oxide, and chitosan, supplemented with N-acetylcysteine	Provided excellent biocompatibility Displayed good anti-hernia and anti-adhesion effects	[93]
Nanofibrous structure	PCL and SF nanofibers embedding multiwalled carbon nanotubes loaded with amoxicillin	Sustained antimicrobial activity against *E. coli* Attenuated the inflammatory response Attenuated foreign body reaction	[94]
Nanofibrous structure	Meshes covered with a nanofibrous membrane made of PCL-gelatin blend	Displayed chemical and topographical cues similar to the native ECM ones	[18]
Stem cells	Solid fiber PCL carrying CTGF and rat mesenchymal stem cells	Avoided multiple complications Provided proper reinforcement	[101]
Composite mesh + stem cells	Composite mesh comprising PLA and a thermo-responsive hydrogel, seeded with rat ADSCs	Provided excellent biocompatibility Could stimulate native ECM Exhibited the capacity to accelerate cell adhesion and proliferation Promoted early vascularization Enhanced defect repair and regeneration	[98]
Composite mesh + stem cells	Composite mesh comprising PP and PCL, seeded with muscle-derived stem cells	Offered good hernia repair, with fewer adhesions and a regenerated muscle layer	[100]
3D printing	PLA mesh composited with a 3D printing of acellular dermal matrix material	Diminished adhesion score Attenuated the inflammatory response Promoted tissue regeneration	[105]
3D printing	Composite mesh comprising PP and PVA loaded with ciprofloxacin hydrochloride	Produced no signs of implant rejection Enabled a faster wound healing Displayed mild to moderate adhesions to the viscera	[106]
3D printing	PCL meshes containing alginate and gentamicin	Exhibited bactericidal activity against *E. coli* Displayed good histopathological behavior	[107]
3D printing + coating	Composite mesh comprising alginate and waterborne polyurethane coated with calcium chloride	Allowed for patient-specific devices Exhibited adequate elasticity for allowing movement	[108]
3D printing + contrast agents	PCL meshes impregnated with iodinated, gadolinium, and barium contrast agents	Displayed excellent visibility on CT	[109]
Heat-shrinkable structure	PLGA heat-shrinkable electrospun fibrous tape	Reduced wound area Ensured tissue repairing	[110]
Polymer blend	Structures fabricated from a PLCL–F-Fg blend in a 4 to 1 ratio	Displayed a balance between material degradation and host tissue in-growth Enabled proper tissue remodeling and reconstruction	[111]
Polymer blend	Mesh fabricated from type I collagen/elastin crosslinked blend	Exhibited similar characteristics to native tissues Promoted tissue restoration Enhanced neovascularization	[116]
Polymer blend + biological matrix	Mesh fabricated from PCL, SF, and micronized decellularized HAM	Stimulated cell proliferation and formation of vasculogenic network Attenuated the inflammatory response Attenuated foreign body reaction Improved incorporation of collagen, elastin fibers, and contractile filaments	[112]
Polymer blend gel	In situ application of an anti-adhesion gel made of oxidized-carboxymethylcellulose-g-dopamine blended with carboxymethyl chitosan	Prevented abdominal adhesions Attenuated the inflammatory response	[27]
Composite material	Composite mesh comprising UPy moieties incorporated into an aliphatic PC backbone	Replaced by a connective tissue stiff enough to prevent herniation Increased inflammatory response Produced signs of muscle atrophy and intramuscular fatty infiltration	[113]
Composite material	Composite mesh comprising poly (ethyl-2) cyanoacrylate reinforced by polyurethane core	Attenuated biological elements for repairing the viscera layer	[114]
Composite material	Composite mesh comprising RGD on the surface and puerarin in the core	Provided excellent biocompatibility Promoted cell viability, adhesion, and proliferation Attenuated the exogenous and endogenous inflammation Stimulated wound healing	[115]
Composite material	Composite mesh comprising a layer of woven cotton fabric modified with gentamicin combined with a market-available PP mesh	Provided excellent biocompatibility Exhibited satisfactory anti-infection properties Strong bactericidal activity against *S. aureus* and *E. coli*	[117]
Composite material	Composite structure comprising a PP mesh covalently bonded with PNIPAAm hydrogel	Exhibited anti-adherent properties	[2]
Design	Tentacle-shaped mesh with a central body and integrated radiating arms	Resulted in a very low rate of complications Greatly reduced the pain	[118]

## Data Availability

Not applicable.
